# *Listeria monocytogenes*-Associated Biliary Tract Infections: A Study of 12 Consecutive Cases and Review

**DOI:** 10.1097/MD.0000000000000105

**Published:** 2014-10-10

**Authors:** Caroline Charlier, Cindy Fevre, Laetitia Travier, Benoît Cazenave, Hélène Bracq-Dieye, Juliette Podevin, Daher Assomany, Lydie Guilbert, Céline Bossard, Françoise Carpentier, Valérie Cales, Alexandre Leclercq, Marc Lecuit

**Affiliations:** Institut Pasteur, Biology of Infection Unit (CC, CF, LT, ML); Institut Pasteur French National Reference Center and WHO Collaborating Center for Listeria (CC, BC, HBD, AL, ML); Inserm U1117 (CC, CF, LT, ML); Université Paris Descartes, Sorbonne Paris Cité, Centre d’Infectiologie Necker-Pasteur, Hôpital Necker-Enfants Malades, Institut Imagine (CC, ML); Service de Chirurgie viscérale, Centre Hospitalier Universitaire de Nantes (JP); Service de Chirurgie viscérale et digestive, Centre Hospitalier de Roubaix (DA, LG); Service d’Anatomopathologie et Cytologie, Centre Hospitalier Universitaire de Nantes (CB); Service d’Anatomopathologie et Cytologie, Centre Hospitalier de Roubaix (FC); and Service d’Anatomopathologie et Cytologie, Centre Hospitalier de Pau (VC).

## Abstract

At present, little is known regarding *Listeria monocytogenes*-associated biliary tract infection, a rare form of listeriosis.

In this article, we will study 12 culture-proven cases reported to the French National Reference Center for *Listeria* from 1996 to 2013 and review the 8 previously published cases.

Twenty cases were studied: 17 cholecystitis, 2 cholangitis, and 1 biliary cyst infection. Half were men with a median age of 69 years (32–85). Comorbidities were present in 80%, including cirrhosis, rheumatoid arthritis, and diabetes. Five patients received immunosuppressive therapy, including corticosteroids and anti-tumor necrosis factor biotherapies. Half were afebrile. Blood cultures were positive in 60% (3/5). Gallbladder histological lesions were analyzed in 3 patients and evidenced acute, chronic, or necrotic exacerbation of chronic infection. Genoserogroup of the 12 available strains were IVb (n = 6), IIb (n = 5), and IIa (n = 1). Their survival in the bile was not enhanced when compared with isolates from other listeriosis cases. Adverse outcome was reported in 33% (5/15): 3 deaths, 1 recurrence; 75% of the patients with adverse outcome received inadequate antimicrobial therapy (*P* = 0.033).

Biliary tract listeriosis is a severe infection associated with high mortality in patients not treated with appropriate therapy. This study provides medical relevance to *in vitro* and animal studies that had shown *Listeria monocytogenes* ability to survive in bile and induce overt biliary infections.

## INTRODUCTION

A severe foodborne infection that mostly occurs in immune-compromised patients is *Listeria monocytogenes* (*Lm*) that is a facultative intracellular Gram-positive bacterium responsible for listeriosis. Three main forms are described: septicemia (S), central nervous system (CNS), and maternal–neonatal (MN) infections. Aside from these typical presentations, localized infections are also reported, mostly as a consequence of a subclinical bacterial systemic dissemination. They include endocarditis, osteoarticular, and cutaneous infections^1–3^ as well as biliary tract infections, which have only been reported as isolated case reports, although *Lm* is well known to colonize the gut and survive in the bile.^4–6^

We undertook a comprehensive retrospective survey over a 17-year period to review all the cases referred to the national surveillance system of listeriosis in France since it has been established. Twelve cases were identified and analyzed. In addition, the 8 previously published case reports were reviewed. This study reveals that among biliary tract infections, those associated with *Lm* tend to exhibit specific features, with a higher frequency of comorbidities, of concomitant bacteremia and of adverse outcome, which are reported in 80%, 60%, and 33% of cases, respectively.^7^ *Lm*-associated biliary tract infections should be considered in the occurrence of biliary tract infection in immunocompromised patients. Their diagnosis requires a clinical and microbiological workup, and treatment is based on a specific amoxicillin-based antibiotic regimen to which *Lm* is sensitive, and which is, otherwise, not recommended as a first-line therapy for biliary tract infections.

## PATIENTS AND METHODS

### Data Collection

Surveillance of human listeriosis in France is based on both mandatory reporting of cases to the Institut de Veille Sanitaire, France, since 1999 and voluntary submission of *Lm* strains to the National Reference Center for *Listeria* (NRCL).^8^ The exhaustiveness of this reporting is estimated above 87%.^9^ We studied all listeriosis cases declared between January 1999 and March 2013 with mention of “cholecystitis,” “cholangitis,” “liver,” or “bile duct.” In addition, all patients with similar clinical data and for whom isolates were sent to the NRCL between 1996 and 1999, before the mandatory reporting era, were also included. Clinicians and microbiologists were contacted, and medical charts were directly analyzed according to a preestablished checklist. An appropriate local ethical committee (Comité de Protection des Personnes Ile de France 8) considered the study as observational and hence exempted to the Institutional Review Board approval, according to the French legislation.

### Review of the Literature

We searched the PubMed database for reports published between January 1966 and June 2013, using the terms “*Listeria*,” “listeriosis,” “cholecystitis,” “cholangitis,” “liver,” and “bile” without language restriction.

### Case Definition

A case was defined as a person from whom *Lm* was isolated from the biliary tract. Infections were classified as cholecystitis, cholangitis, or biliary tract cyst infection. Liver abscesses without bile tract infection were excluded. Diagnosis of concurrent septicemia was based on a positive blood culture.

### *L monocytogenes* Typing

*Listeria* isolates referred to the NRCL were identified with API *Listeria* (BioMérieux, Marcy l’Etoile, France), serotyped until January 2005,^10^ and then typed by multiplex polymerase chain reaction (PCR) genoserogrouping.^11^ PCR serogroups correspond to the 4 major serovars that cause human disease.^11^ Isolates were characterized by multilocus sequence typing (MLST) similar to the 745 other strains received in the NRCL, as previously described.^12–14^

### Bile Resistance Assays

Forty-two isolates were tested: 10 from biliary tract infection referred to the NRCL, the EGD bile susceptible, the LO28 bile-resistant reference strains,^6^ and 30 clinical isolates randomly selected among those received by the NRCL in 2012 (10 for each form: S, CNS, and MN). All were grown to log phase in brain heart infusion (BHI) broth (Becton Dickinson, Le Pont de Claix, France) at 37°C overnight, and then were inoculated by a A400 Multipoint Inoculator (Denley, West Sursex, UK) yielding 10^4^ cells per spot on pork bile agar plates containing BHI base medium and 0%–10%, 15%, 20%, 25%, and 30% pork bile (Sigma Chemical Co, St Louis, MO) (pH = 7). The plates were incubated at 37°C in anaerobic condition for 24 hours and the minimal inhibitory concentration (MIC) of bile, defined as the lowest concentration totally inhibiting the growth of spots, was determined for each isolate. Mann–Whitney test was used to compare MICs.

### Indirect Immunofluorescence Assay of InlA Surface Expression

Bacteria were grown overnight in liquid BHI at 37°C, rinsed, incubated with I4.4/L7.7 monoclonal antibodies against InlA (1:1000)^15^ for 1 hour, and then with a secondary goat anti-mouse Alexa Fluor 488 antibody (Life technologies, Carlfbad, CA) (1:500). Preparations were analyzed by epifluorescence microscopy (AxioObserver Z1 inverted microscope, Zeiss) and analyzed with the AxioVision software (Zeiss). *Lm* EGD that expresses surface InlA was used as positive control and *Lm* L028 that expresses a secreted truncated InlA was used as negative control. In case of absence of InlA surface expression, InlA truncation was confirmed by *inlA* sequencing.^12^

### Biofilm Assays

Forty-four isolates were tested: 12 bile tract infection isolates referred to the NRCL, the EGD, the LO28 *Lm* reference strains,^6^ and the 30 clinical isolates described above. Cultures were performed in BHI broth at 37°C upon shaking. Aliquot of BHI overnight liquid cultures (1:20) was added to fresh BHI medium. Exponential cultures were diluted in BHI medium, BHI with pork bile, at pH 5 and 7, to an optical density (OD_600 nm_) of 0.06 in 100 μL 96-well poly (vinyl chloride) microtiter plates (Falcon; Becton Dickinson Labware, Oxnard, CA). Biofilms were allowed to grow for 24 hours at 37°C. Unbound cells were removed by microplate inversion and tapping on absorbent paper. Microplates were washed in water and adherent cells were stained with crystal violet for 20 minutes. Excess stain was removed by 3 washes in water. Quantification of bound cells was performed by adding acetone–ethanol (20:80) and dissolved crystal violet was measured at OD_595 nm_. Each biomass was standardized relative to EGD reference strain, and Mann–Whitney test was used to compare each group.

### Histopathological Analyses

Eight-micrometer-thick sections of paraffin-embedded tissue specimens were stained with hematoxylin eosin. *Lm* was labeled by immunohistochemistry using a polyclonal rabbit antiserum that detects *Lm* serotype 4b (Listeria O V/VI antiserum Seiken kit; Denka Seiken Co, Tokyo, Japan) and a goat anti-rabbit antibody coupled to peroxidase (EnVision+, Dako, Glosturp, Denmark), followed by hematoxylin counterstaining. Images were captured on a AxioImager A2 microscope (Zeiss) equipped with an AxioCam ICc 1 digital camera (Zeiss) and the AxioVision 4.8 software (Zeiss).

## RESULTS

### Clinical Cohort

A retrospective analysis of all cases declared to the NRCL was performed as described in the “Patients and Methods” section. Among the 3231 human cases for which a clinical *Lm* strain was collected between January 1996 and March 2013, 12 involved patients with biliary tract infections (hereafter named the French cohort), representing 0.37% of the infections reported during the study period. They included 9 cholecystitis (75%), 2 cholangitis (17%), and 1 biliary cyst infection (8%) that is listed in Table 1  .^16–22^ Eight additional cases were identified in the literature; all were cholecystitis and are also listed in Table 1  . The patients from the French cohort and those previously reported were analyzed together to identify the main characteristics of *Lm*-associated biliary tract infections.

**TABLE 1:**
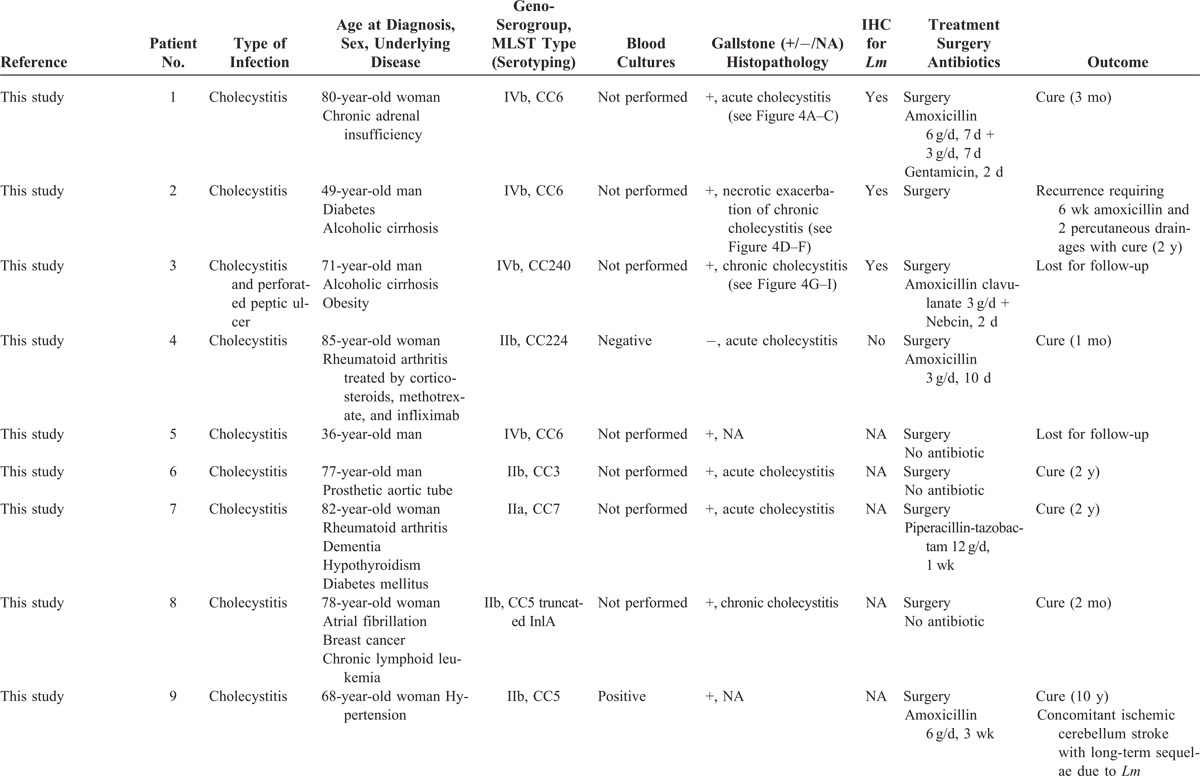
Characteristics of 20 Patients With *Lm*-Associated Biliary Tract Infections

**TABLE 1 (Continued):**
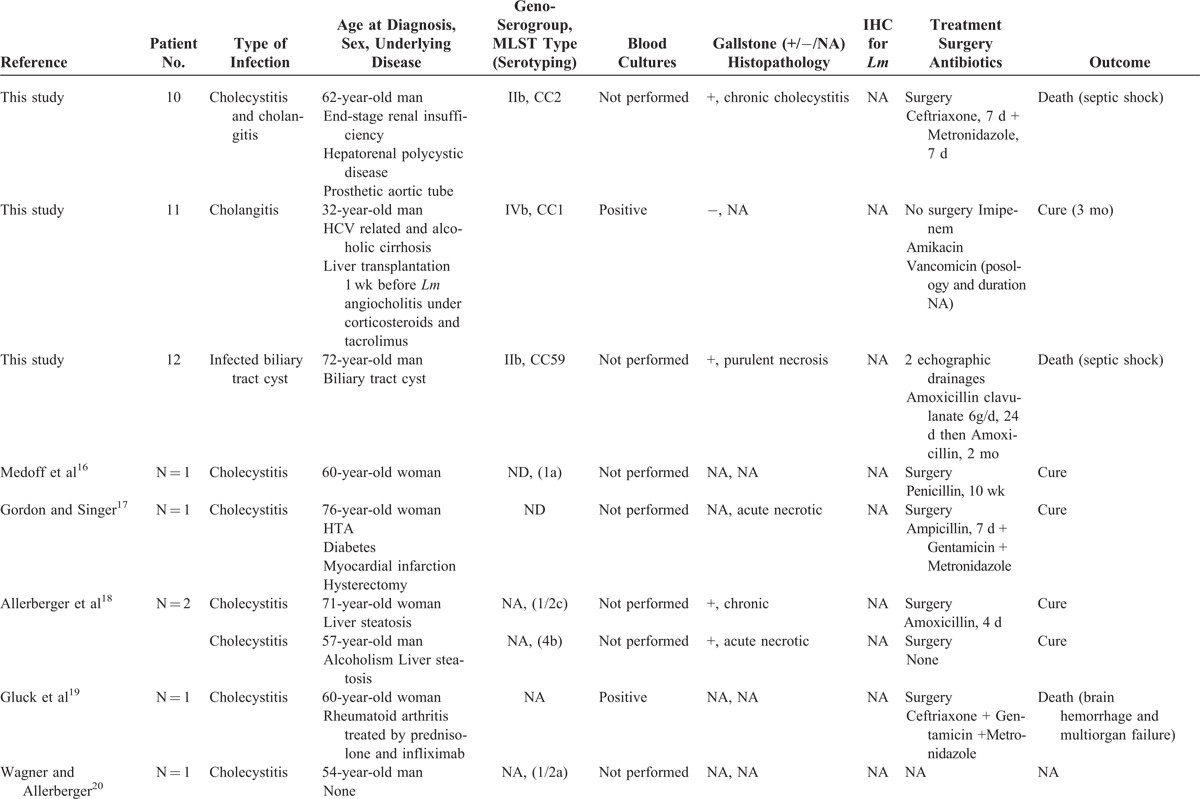
Characteristics of 20 Patients With *Lm*-Associated Biliary Tract Infections

**TABLE 1 (Continued):**
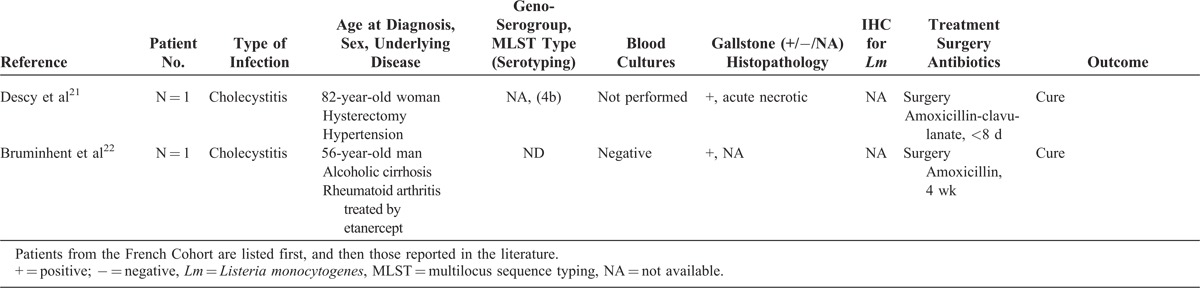
Characteristics of 20 Patients With *Lm*-Associated Biliary Tract Infections

### Epidemiology

Ten patients were men (50%) and their median age was 69 years (range 32–85). Comorbidities are detailed in Table 1  : 16 patients (80%) had 1 to 4 associated comorbidities (16/20), which included cirrhosis, hypertension, and rheumatoid arthritis (n = 4, each), diabetes (n = 3), aortic patch tube (n = 2), obesity, end-stage renal insufficiency, liver transplantation, chronic adrenal insufficiency, myocardial infarction, dementia, hypothyroidism, chronic obstructive pulmonary disease, chronic lymphoid leukemia, and breast cancer (n = 1, each). Five patients were receiving immunosuppressive drugs at the time of *Lm*-associated biliary tract infections, namely, corticosteroids (n = 4), infliximab plus methotrexate (2/17, 12%, one of them with additional ciclosporin), etanercept, tacrolimus, and fludarabin (n = 1, each). Cholecystolithiasis was reported in 88% of the patients with cholecystitis (14/16).

### Clinical Features

Median time from first symptom to hospitalization was 2 days (range 0–60, n = 12) with all but 2 patients hospitalized within the first week of symptoms. Median duration of hospitalization was 11 days (range 1–96, n = 18). Fever was reported in 50% of the cases (10/20, range 38–40°C). Abdominal pain was reported in 88% of cases (15/17). Previous or concomitant diarrhea and vomiting were observed in 11% (2/18). Gastric ulcer was concomitantly diagnosed in 2 other patients (2/18, 11%). Jaundice was noted in only 1 case with cirrhosis (1/18, 6%). None of these cases arose in the context of neurolisteriosis or pregnancy-associated listeriosis.

### Laboratory Characteristics

Transaminases blood levels ranged from <1N to 6N (n = 15), and median aspartate amino transferase and alanine amino transferase were 73 and 49 UI/mL, respectively. Total bilirubin blood level was normal in 93% of cases (13/14). Median leukocytosis was 8100/mm^3^ (range 4200–17,500, n = 17), with only 2 patients with leukocytosis above 12,000/mm^3^. Median lymphocyte count was 1221/mm^3^ (range 432–3690, n = 10), including 3 patients below 1000/mm^3^. Median hemoglobin blood level was 14 g/dL (n = 15), and median platelet count was 189,500/mm^3^ (n = 14) including 3 patients below 100,000/mm^3^. Median C-reactive protein blood level was 125 mg/L (range 11–300, n = 8).

### Microbiological Features

Diagnosis was confirmed by bile or gallbladder swab culture in all the cases. *Lm* was never suspected before culture results and was the only recovered pathogen in all cases. Blood cultures were performed in only 5/18 patients, 4 of them had temperature >38°C; they were positive in 3 (60%). Further microbiological analyses were performed on the 12 French isolates. Antimicrobial sensitivity was unremarkable when compared with a large panel of more than 4000 clinical strains.^23^

#### Genoserogrouping

French strains were collected from patients originating from various geographical origins, at various times (1996, 1997, 1998, twice in 1999, 2000, 2003, 2008, 2009, twice in 2010, and 2013). They belonged to 3 major genoserogroups: IVb (n = 6/12), IIb (n = 5/112), and IIa (n = 1/12), and matched the overall distribution of human clinical isolates in France during the same period (data not shown).

#### MLST

**FIGURE 1:**
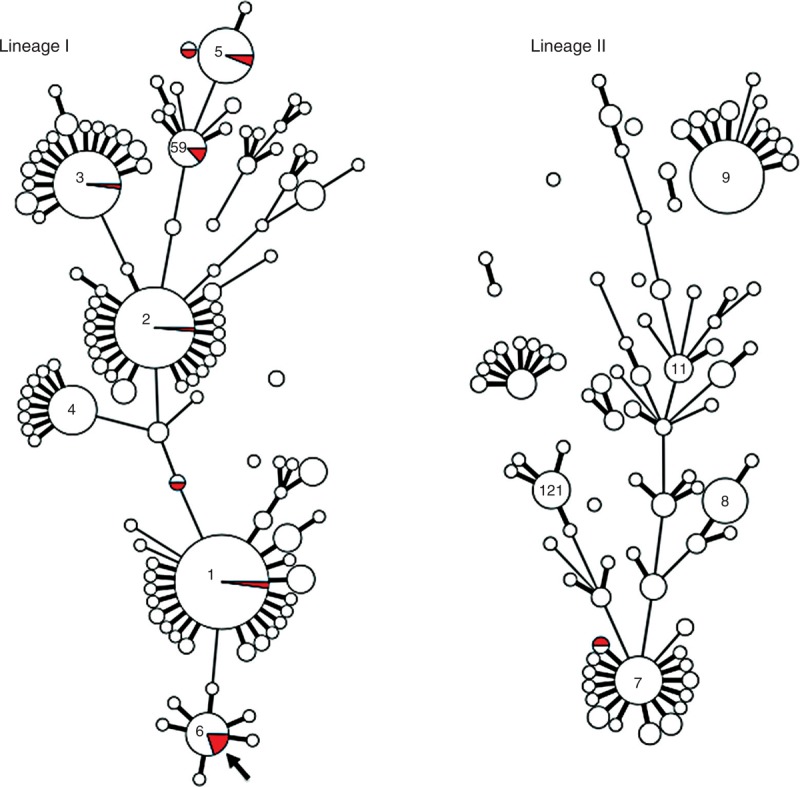
Multilocus sequence typing-based minimum spanning tree of 745 *Listeria* *monocytogenes* isolates of lineages I and II. Each circle denotes a single type (ST) and the diameter reflects the number of isolates in that ST. Red sectors denote biliary-tract infections isolates; white sectors correspond to other isolates. Bold lines between circles correspond to links with a single allelic mismatch; plain lines correspond to those with 2 allelic mismatches. Links corresponding to more than 2 allelic mismatches are not represented, as several equally likely alternative links exist; therefore, the relative positions of clonal complexes (CCs) or single STs should not be taken as evidence of phylogenetic proximity. Values inside circles indicate the ST numbers of the central STs of numerically important CCs. Left panel represents lineage I whereas right panel represents lineage II. The arrow denotes 2 isolates from the same patient (Patient 2) who presented with a documented recurrence of infection. All data are available at http://www.pasteur.fr/mlst.

Th strains were identified as belonging to 9 distinct clonal complexes (CCs), without any CC associated with biliary tract strains (Figure 1).

#### InlA Surface Expression

Among the 12 strains of the French cohort, 1 exhibited a truncated form of InlA, confirmed by sequencing (data not shown).

#### Bile Survival Assays

**FIGURE 2:**
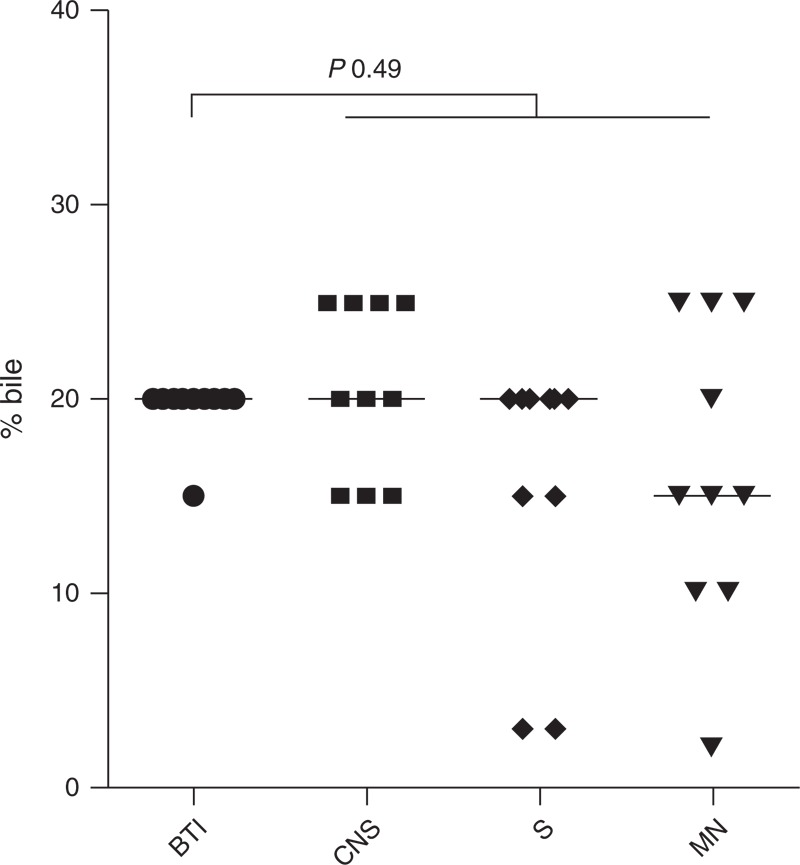
Bile resistance in 10 French isolates and in 30 strains selected at random among isolates received in 2012 from the National Reference Center for *Listeria* from patients with septicemia (S), central nervous system (CNS), and maternal–neonatal (MN) infections. The minimal inhibitory concentration of bile for a strain was interpreted as the lowest concentration totally inhibiting the growth of spots. *P* values were determined as compared to S, CNS, and MN isolates (Mann–Whitney test). BTI = bile tract infections.

MICs were similar for *Lm* biliary tract isolates and from S and CNS (20%, *P* > 0.05) (Figure 2).

#### Biofilm Assays

**FIGURE 3:**
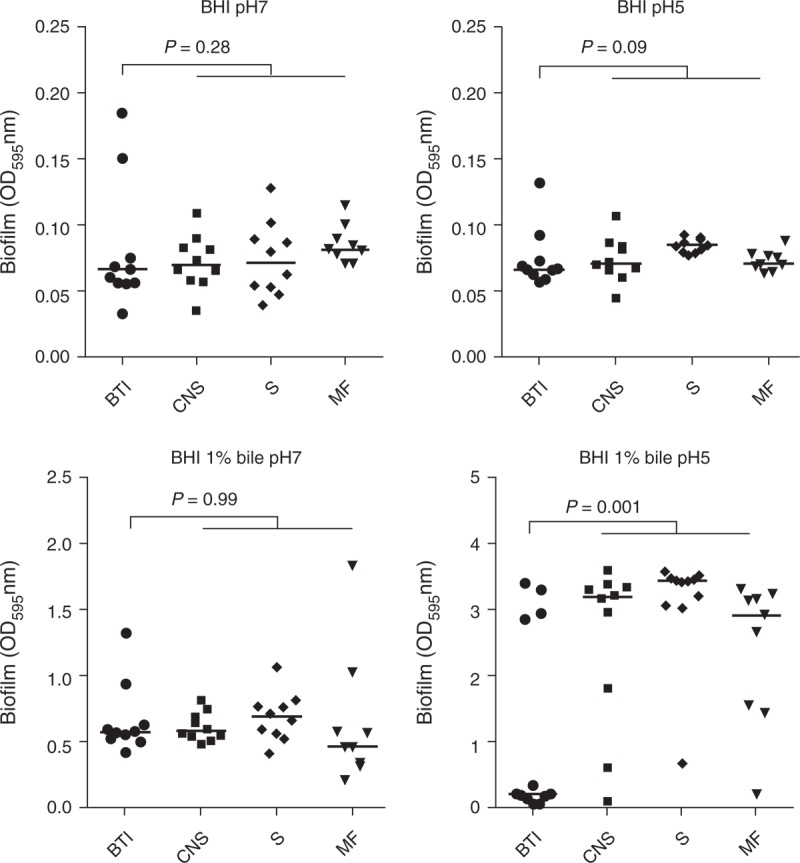
Biofilm formation in 12 French isolates and in 30 strains selected at random among isolates received in 2012 from the National Reference Center for *Listeria* from patients with septicemia (S), central nervous system (CNS), and maternal–neonatal (MN) infections. *P* values were determined as compared to S, CNS, and MN isolates (Mann–Whitney test). BHI =  brain heart infusion, BTI = bile tract infections.

No difference in biofilm ability among strains was observed with or without pork bile at pH 7 reflecting the gallbladder conditions. In the presence of pork bile, at pH 5 reflecting duodenal conditions, bile tract isolates had significantly lower biofilm ability than those from S, CNS, and MN infections (*P* = 0.001) (Figure 3).

### Histology

**FIGURE 4:**
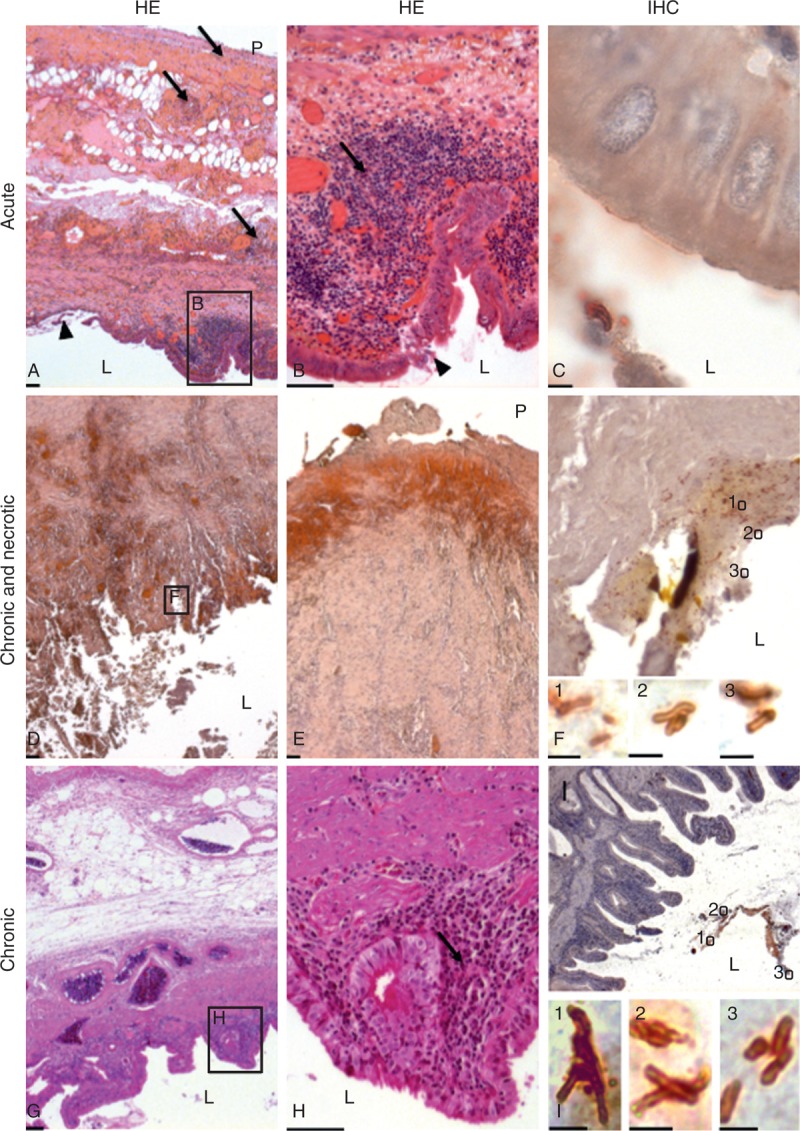
(A–C) Gallbladder sections from 3 patients with acute cholecystitis (Patient 1), (D–F) necrotic exacerbation of chronic cholecystitis (Patient 2), and (G–I) chronic cholecystitis (Patient 3). *Lm* was genoserotyped as IVb in the 3 cases. In acute cholecystitis, HE staining revealed edematous congestive transmural inflammation (A; arrow), polymorphonuclear cells infiltrate (B; arrow), and focal mucosal ulceration (B; arrowhead). *Lm* was seen in the lumen as aggregates and individual bacteria (C). In the necrotic exacerbation of chronic infection, necrosis involved the mucosa and the muscularis (D, E), with necrotic luminal tissue debris and inflammatory fibrosis in the serosal coat. *Lm* was located in the lumen and in necrotic tissues lining the lumen (F). In chronic cholecystitis, diffuse mucosal-based infiltrate of mononucleate cells was observed (G, H; arrow). *Lm* was observed inside the lumen (I). Scale bars: HE staining, 100 µm; IHC staining, 2 µm. HE = hematoxylin–eosin, IHC = immunohistochemistry, L = lumen, P = peritoneal cavity.

Gall bladder histopathology was analyzed and anti-*Lm* immunoenzymatic labeling was performed in 3 patients for whom gallbladder samples were available (Table 1  ). Cholecystitis with cholecystolithiasis was confirmed in all the cases (Figure 4). Patient 1 had acute cholecystitis with edematous congestive transmural inflammation (Figure 4A), polymorphonuclear cells infiltrate (Figure 4B), and focal mucosal ulceration. Patient 2 had necrotic exacerbation of chronic cholecystitis (Figure 4D), with necrosis of the mucosa and muscularis (Figure 4D and E), necrotic luminal tissue debris, and inflammatory fibrosis in the serosal coat. An aspect evocative of chronic cholecystitis was observed in Patient 3, with a diffuse mucosal-based infiltrate of mononucleate cells (Figure 4G and H). Such patterns of acute, chronic, and necrotic exacerbations of chronic infection mirror those reported in classical cholecystitises. The presence of *Lm* was confirmed in 3 cases (Figure 4C, F, and I). Bacteria were located in the gallbladder lumen, sometimes as aggregates (Figure 4C and I), or within tissue fragments in necrotic cholecystitis (Figure 4F).

### Treatment and Outcome

Surgical and medical treatments are detailed in Table 1  . All patients with cholecystitis underwent cholecystectomy, and all those with cholangitis or collection had drainage of infected bile. Sixty-five percent of patients (13/20) had penicillin-based regimens with various dosages and durations. Three of them received aminoglycosides. Inadequate therapy with cephalosporin (n = 2) or no antibiotic (n = 5) was reported in 35% (7/20). Five patients were lost for follow-up. Adverse outcome was reported in 33% (5/15), namely, 3 early deaths, 1 microbiologically proven recurrence (see case report and Table 1  ), and 1 concomitant cerebellous stroke unrelated to *Lm* (Table 1  ). Of the 4 patients who died or experienced microbiological failure, 3 (75%) did not receive any antibiotic (n = 1) or were treated by inappropriate cephalosporin-based regimens ineffective against *Lm* (n = 2). Inadequate treatment (ineffective or lack of thereof) was significantly associated with the occurrence of death or microbiological failure, defined as a microbiologically proven recurrence (*P *= 0.033, Fisher exact test).

## DISCUSSION

Here, we have studied the detailed features of *Lm*-associated biliary tract infections in a cohort of 20 cases, that includes 12 new consecutive cases declared in France over the last 17 years and 8 previously published reports. Important conclusions can be drawn from this study. First, *Lm*-associated biliary tract infection is a genuine clinical entity. It mostly involves older patients with comorbidities and is associated with a much higher mortality rate than other biliary tract infections (15% vs 3%, *P* < 0.03).^24^ The nonprescription of amoxicillin/ampicillin or other effective antibiotics such as ampicillin/sulbactam, piperacillin/tazobactam, or carbapenems might be associated with poor outcome: death or recurrence of infection (*P* = 0.03). These conclusions have important implications for clinicians: the identification of *Lm* in a bile sample should lead to the swift prescription of amoxicillin/ampicillin, which should be maintained beyond the perioperative period, in contrast to current guidelines for the management of community-acquired biliary tract infections that recommend discontinuation of antibiotics within 24 hours after cholecystectomy in the absence of infection outside the gallbladder wall.^25^ Among the first-line drugs recommended in uncomplicated community-acquired cholecystitis, third-generation cephalosporins should not be used because of their intrinsic lack of activity, whereas carbapenems and piperacillin/tazobactam both display bactericidal activity toward *Lm*. Peroperative bacteriological sampling is not systematically recommended and the absence of fever reported in most cases does not lead to the prescription of blood cultures: this likely leads to an underestimation of the actual burden of biliary tract infections associated with *Lm*. The main limitation of the study is its retrospective nature, because of the rarity of the disease.

*Lm* is known to colonize the gut and *Lm* asymptomatic fecal carriage has been documented in 1% to 12% of healthy individuals.^26–28^ Bile exhibits antimicrobial activities, given its ability to interact with membrane lipids and damage bacterial membranes.^29^ *Lm*, as many other enteric pathogens, has evolved to survive in the bile and in the proximal region of the small intestine where bile is released.^30^ The occurrence of biliary tract infections associated with *Lm* is therefore not surprising. Indeed, all *Lm* strains express a bile salt hydrolase encoded by *bsh* that detoxifies bile by deconjugating glycine/taurine from bile salts.^6^ *Lm* is also able to accumulate solutes such as betaine and carnitine, thereby enhancing its resistance to stress conditions,^31^ and the osmolyte transporters OpuC, BetL, and Gbu involved in their uptake play a major role in *Lm* tolerance to the bile.^4,5^ An active bile exclusion system called BilE is also implicated in *Lm* survival in bile.^5^ All these systems are transcriptionally regulated by PrfA, *Lm* master virulence gene regulator, and are functionally active at the low pH of the proximal small intestine. Other genes and metabolic pathways implicated in amino acid synthesis, purine metabolism, and biotin uptake have been more recently identified in *Lm* and may be involved in resistance to bile stress in gallbladder neutral pH conditions.^32^

Consistent with these *in vitro* data, *Lm* is able to colonize the gall bladder after both oral and intravenous challenge in a mouse model of infection.^30^ It can survive and multiply extracellularly in the mouse gallbladder lumen, be released via the biliary tract in the intestinal lumen, and induce overt cholecystitis.^30,33,34^ These experimental findings match those observed in the present cohort of patients with *Lm*-associated biliary tract infection (Figure 4) and are therefore relevant to the human situation. Indeed, as observed in the mouse, *Lm* was consistently found extracellularly in the gallbladder of the patients (Figure 4). Moreover, 1 of the clinical strains expresses a truncated and, therefore, nonfunctional form of InlA unable to mediate *Lm* internalization, further illustrating that *Lm*-associated biliary tract infection does not result from epithelial invasion, a finding that is also *de facto* observed in the mouse model of biliary tract infection, InlA being not functional in the mouse.^30,35^

Survival in bile *in vivo* and the ability to induce biliary tract infection is likely a general property of *Lm* for several reasons. First, the isolates responsible for *Lm*-associated biliary tract infection do not belong to specific clonal complexes but reflect the diversity of the strains isolated from patients with listeriosis. Second, biliary tract infection isolates do not have increased survival in bile-containing medium. They do not either exhibit enhanced biofilm-producing ability, including in a bile-rich environment.

The ability of *Lm* to survive in bile has several clinical consequences. First, asymptomatic *Lm* bile colonization could be nonpathogenic *per se*, but serve as a reservoir reinoculating the proximal small intestine where *Lm* crosses the intestinal barrier.^36,37^ It could also constitute the reservoir that feeds long-term fecal shedding, reported in up to 12% of patients. This would facilitate dissemination and have major public health implications, as described for *Salmonella enterica* serovar Typhi.^38^ Although not formally demonstrated in humans, this sequence of events has been observed in mice, where bioluminescence studies have shown the release of viable *Lm* in the bile during gallbladder contractions and their expulsion in the digestive tract. From the gut, they could reinfect the host and disseminate into the environment.^39^ Finally, obstructing lithiasis in the context of preexisting *Lm* bile colonization is the most probable trigger of overt *Lm* cholangitis/cholecystitis, as described in other typical biliary tract infections.^7^

The lower positivity rate of blood cultures compared with the bile cultures mirrored previously published data on bile tract infections.^40^

Microbiological examination of the bile/gallbladder and blood cultures are far from being routinely performed in cholecystectomized patient with cholecystitis, and *Lm*-associated biliary tract infection, although certainly rare, therefore likely remains largely undetected in the clinical practice. Furthermore, piperacillin/tazobactam is routinely used in the United States to treat patients with biliary infections and may explain the rare isolation of *Listeria*. In a review compiling 211 cases of cholecystectomy (including 34 urgent and 177 elective surgeries), diphteroid-like rods compatible with *Lm* were evidenced in at least 3 cases,^41^ yet no further characterization was performed. As culture-based pathogens detection could be lowered by preoperative prophylaxis (http://www.sages.org/publication/id/06/), more recent studies using PCR tools have also been performed to identify pathogens involved in cholecystitis. Neither Lemos et al,^42^ in a Brazilian study involving 84 patients who had not receive preoperative antibioprophylaxis, nor Lee et al,^43^ in a Korean study performed on bile tract samples from 156 patients, evidenced any *Lm*.

*Lm* biliary tract infection should be considered as a genuine although rare cause of cholecystitis. In contrast, the occurrence of transient and asymptomatic *Lm* bile colonization could be frequent although this remains to be established in the context of prospective studies.

In conclusion, the results from this study validate in human the experimental data that had been obtained in the mouse and provide strong evidence that the presence of *Lm* in the bile should be taken into account by clinicians. *Lm-*associated biliary tract infection requires a specific treatment based on surgery and the prescription of amoxicillin. *Lm* survival in the bile and chronic colonization in the biliary tract is not only a cause of morbidity and mortality. The biliary tract also likely constitutes a reservoir that favors *Lm* long-term fecal carriage and transmission.
